# Citizen Chaussier, on Amputation

**Published:** 1800-11

**Authors:** 


### *Citizen* Chaussier, *on Amputation.*

In the Philomathic Society of Paris, Cit. Chauffier has read a Memoir, containing Experiments on the Amputation of the articulating Extremities of the cylindrical Bones.

As this Memoir contains fome interefting anatomical and phyfiological facts, we fhall prefent our readers with fome of the particulars. Several furgeons of eminence, both in England and France, have propofed and occafionally practifed an excifion of the articulating proceffes in difeafes of the larger joints, inftead of amputating the whole limb, and this practice has fometimes preferved to the patient, a limb which has afterwards become of confiderable ufe. It was in order to determine mine the practicability of this operation, that M. Chauffier has made feveral experiments upon animals, of which the following are the refults:

Having laid bare by incifion the upper extremity of the femur, he turned the head of the bone out of its articulation, and fawed it off, fome way below the trochanter; fo as to take away an eighth, a fixth, and fometimes a fourth of the whole length of the bone. He then brought together the mufcles and fkin, fecured their union by a few futures, and left the reft to Nature. The wounds always clofed without fuppuration, or any apparent exfoliation of the bone, and the cicatrix was compleatly formed at the tenth, or, at lateft, the fifteenth day. In a month's time the animal began to make fome ufe of the paws of the limb operated upon.

By examining at different periods the parts that had fuffered the operation, it was found that the mufcles had, by their contraction, forced the upper part of the femur upon one of the projecting points of the ifchium; that the amputated extremity was rounded off, and covered with a cartilaginous fubftance; that the part of the ifchium upon which it had been thrown, had alfo affumed a cartilaginous appearance, and fometimes formed an articular *fossa*, more or lefs deep; that the cellular fubftance around this new articulation, produced a kind of capfular membrane which contained a ferous liquor; and the acetabalum gradually became filled up with cellular fubftance.

In order to afcertain what changes fuppuration would produce in this procefs, the experiment was repeated upon a dog; but inftead of promoting the union of parts by the firft intention, the wound was irritated in feveral ways, and fuppuration brought on.

The animal fuffered much, and feveral abfceffes were formed, which opened fucceffively. However, after two months he was compleatly cured, and retained a very good ufe of the foot, After four years the parts were examined, and it was found that the extremity of the femur was attached to the ifchium by a ligamento-cartilaginous fubftance, which fixed it upon this bone, and allowed of free motion in every direction. There was alfo formed at the extremity of the femur an apophyfis, which gave attachment to the different mufcles, and ferved inftead of a trochanter.

The fame operation, performed on the upper extremity of the humerus, had the fame fuccefs, and gave very nearly the fame refults.

M. Chauffier repeated thefe experiments on the *lower* extremity of the femur, the cubital joint of the humerus, and the lower part of the tibia; and he even, in fome cafes, took away the whole knee and elbow joints of different animals; but though none of them died, the operation never fucceeded: The foft as well as the hard parts healed up well; but inftead of forming a new articulation, the extremities of the upper and lower bones receded from each other, and the part below the articulation remained loofe and pendulous, and entirely ufelefs to the animal. Thefe operations on ginglymus joints are, befides, very difficult to perform, and attended with much hazard on account of the arterial ramifications; and could promife no fuccefs in real practice, on account of not being furrounded and covered with any great quantity of flefh.

At the conclufion of this Memoir, M. Chauffier mentioned a curious cafe of a young man, with whom the upper extremity of the humerus feparated fpontaneoufly, after a caries of the bone and tedious ulceration, which had at laft been cured by the efforts of Nature. A new and very remarkable articulation had been formed in this cafe. The fcapula fent out a rounded protuberance like the head of a bone, and the humerus had a correfponding cavity which received it, and thus allowed the patient almoft every ufe of his arm, as in its natural ftate.

